# Molecular Basis for Endocrine Disruption by Pesticides Targeting Aromatase and Estrogen Receptor

**DOI:** 10.3390/ijerph17165664

**Published:** 2020-08-05

**Authors:** Chao Zhang, Tiziana Schilirò, Marta Gea, Silvia Bianchi, Angelo Spinello, Alessandra Magistrato, Gianfranco Gilardi, Giovanna Di Nardo

**Affiliations:** 1 Department of Life Sciences and Systems Biology, University of Torino, 10123 Torino, Italy; chao.zhang@unito.it (C.Z.); silvia.bianchi.04@hotmail.it (S.B.); gianfranco.gilardi@unito.it (G.G.); 2 Department of Public Health and Pediatrics, University of Torino, 10126 Torino, Italy; tiziana.schiliro@unito.it (T.S.); marta.gea@unito.it (M.G.); 3 National Research Council-Institute of Materials (CNR-IOM) at International School for Advanced Studies (SISSA), 34165 Trieste, Italy; angelo.spinello@sissa.it (A.S.); alessandra.magistrato@sissa.it (A.M.)

**Keywords:** aromatase, estrogen receptor, endocrine disrupting chemical, pesticides, neonicotinoids, estrogenic activity, gene reporter assay, MELN allosteric inhibition, molecular dynamics

## Abstract

The intensive use of pesticides has led to their increasing presence in water, soil, and agricultural products. Mounting evidence indicates that some pesticides may be endocrine disrupting chemicals (EDCs), being therefore harmful for the human health and the environment. In this study, three pesticides, glyphosate, thiacloprid, and imidacloprid, were tested for their ability to interfere with estrogen biosynthesis and/or signaling, to evaluate their potential action as EDCs. Among the tested compounds, only glyphosate inhibited aromatase activity (up to 30%) via a non-competitive inhibition or a mixed inhibition mechanism depending on the concentration applied. Then, the ability of the three pesticides to induce an estrogenic activity was tested in MELN cells. When compared to 17β-estradiol, thiacloprid and imidacloprid induced an estrogenic activity at the highest concentrations tested with a relative potency of 5.4 × 10^−10^ and 3.7 × 10^−9^, respectively. Molecular dynamics and docking simulations predicted the potential binding sites and the binding mode of the three pesticides on the structure of the two key targets, providing a rational for their mechanism as EDCs. The results demonstrate that the three pesticides are potential EDCs as glyphosate acts as an aromatase inhibitor, whereas imidacloprid and thiacloprid can interfere with estrogen induced signaling.

## 1. Introduction

Under the modern lifestyle, humans are exposed to various chemicals such as pesticide residuals in fruits and vegetables, antibiotics in meat and milk, preservatives in cosmetics and personal care products [1,2]. These chemicals are usually in low doses and may not have a short term significant impact on the human body, but they can cause long term damages to health [3]. The effects of low-dose compounds on human health are mainly related to the endocrine system [4,5,6]. These compounds can in fact mimic or influence the action of endogenous hormones through various mechanisms, being therefore referred to as endocrine disrupting chemicals (EDCs) [7,8].

The modern industrial and agricultural system relies heavily on pesticides. The compelling need of high food crop increasingly demands the use of chemicals. This results in the extensive applications of millions of tons of pesticides every year [9,10]. Among the many pesticides available on the market, glyphosate is one of the most widely used herbicides in the world. In plants, it affects the synthesis of essential aromatic amino acids by inhibiting the activity of 5-enolpyruvylshikimate-3-phosphate synthase (EPSPS) in the shikimate pathway [11]. As a result, glyphosate is considered harmless to mammals since they do not contain the EPSPS enzyme [12]. Conversely, neonicotinoids are currently the most widely used agricultural insecticides [13]. These selective insecticides specifically bind to the α -subunit of nicotinic acetylcholine receptors (nAChR), which is common in all insects [14,15]. Due to their difficulty to penetrate the blood-brain barrier, they should exert low toxicity in vertebrates. However, different direct, indirect, and suspect toxic effects of these compounds on vertebrate wildlife and human health have been reported [16,17,18]. Neonicotinoids were introduced in the market in the 1990s, therefore their sales volume has enormously proliferated in recent decades, exceeding 25% of the market in 2010 [19]. Imidacloprid has become the world’s best-selling insecticide next to glyphosate [20]. Although the European Union banned the outdoor application of imidacloprid, clothianidin, and thiamethoxam in 2018 due to a high risk for bees that are responsible for pollinating most crops worldwide [21], neonicotinoids remain the most extensively applied insecticides in the world [22].

The intensive use of glyphosate-based herbicides and neonicotinoid insecticides has caused the contamination of soil, water, air, and agricultural products [23,24,25,26,27,28]. The half-life of glyphosate in the field is usually 47 days and it is primarily degraded into aminomethylphosphonic acid (AMPA) and glyoxylic acid by soil microorganisms [29]. Neonicotinoids can exist for a long time in soil and water, and their half-life being as long as three years [22]. Given these observations and the increased use of pesticides, concerns are raising about their potential impact on human health and the environment. In particular, mounting evidence discloses that exposure to pesticides can affect the endocrine system. Toxicological and epidemiological studies indicate possible genotoxic and cytotoxic effects as well as birth defects and neurotoxicity in different cell lines and animal models [18,30].

Aiming at establishing the interference of pollutants with human health, we monitored the effect of glyphosate and two neonicotinoids on two critical targets of the endocrine system: Aromatase, the enzyme responsible for estrogen biosynthesis, and estrogen receptor (ER) alpha, the main protein promoting estrogen signaling.

Aromatase catalyzes the transformation of androgens to estrogens [31]. In vitro experiments showed that glyphosate causes changes of aromatase mRNA levels and activity in placental JEG3 cells and human HEK293 cells, thus interfering with steroid conversion to estradiol [32,33]. In addition, the expression of androgen and ERα was inhibited in glyphosate treated HepG2 cells [34,35] where also the transcription and activity of aromatase were altered [35]. Insecticides such as thiacloprid, thiamethoxam, and imidacloprid affect aromatase expression and activity in a co-culture model of fetoplacental steroidogenesis, increasing estrone and estradiol production, while estriol production is decreased [36]. Thiacloprid, imidacloprid, and thiamethoxam have also been demonstrated to increase aromatase expression and activity in H295R and Hs578t cells [37,38].

Here, a recombinant form of human aromatase is used to test the possible effect of the three pesticides on estrogen biosynthesis, gaining new information at molecular level [39,40,41]. Moreover, all-atom molecular dynamics simulations provided structural insights on the ability of these molecules to target the aromatase enzyme.

At a cellular level, the estrogen-regulated signaling is mainly due to estrogen receptors (ERs). The main ERs are the ERα and ERβ nuclear receptors and the G-protein-coupled estrogen receptor 1, which is a membrane receptor [42]. Estrogen receptor α (ERα) is a nuclear hormone receptor and a ligand-regulated transcription factor, which mediates the activity of estrogens in vital processes (i.e., reproduction, cardiovascular maintenance, bone density/remodeling). ERα is composed of five functional domains, among which the ligand-binding domain, activated upon estrogen binding, stimulates cell growth and proliferation. After the menopause, increased estrogen levels due to a deregulated activity of aromatase bind as an agonist to ERα, exerting a pro-oncogenic effect by either decreasing apoptosis or promoting cell proliferation [43]. Therefore, estrogen selective modulators have been developed and one of them, tamoxifen, is extensively used in adjuvant therapy of breast cancer for its ability to act as an ERα antagonist.

Previous studies have investigated the ability of glyphosate to induce an estrogenic activity mediated by ERs. The results of the study of Thongprakaisang et al., 2013 [44] demonstrated that glyphosate induces an ER-mediated estrogenic activity, mediated by ER activation, similar to 17β-estradiol (E2) on T47D-KBluc cells. However, recently another study, using the same cells, showed that this pesticide induces an estrogenic activity, mediated by ER activation, lower than E2 and that this activation is probably induced by a ligand-independent mechanism [45]. Moreover, additional studies on different transfected cells showed that glyphosate did not induce any ER-mediated estrogenic activity and did not produce any anti-estrogenic effect when tested in combination with E2 [35,46]. Therefore, whether glyphosate can trigger an ER-mediated estrogenic activity remains controversial. To our knowledge, only three studies have been performed on the estrogenic activity of imidacloprid and thiacloprid. In particular, in the study of Mesnage et al., 2018 [47] the proliferative effect of both pesticides was investigated on estrogen-sensitive cells, while Kojima et al., 2004 [46] and Westlund and Yargeau, 2017 [48] assessed the ER-mediated estrogenic activity of imidacloprid and thiacloprid on mammalian or yeast cells, respectively. The results of the three studies demonstrated that the two pesticides induce no proliferative effect and no estrogenic activity was mediated by ERs, while an anti-estrogenic activity was detected testing imidacloprid in combination with E2 on yeast cells.

In this work, in order to increase the knowledge on the estrogenic activity of glyphosate, imidacloprid, and thiacloprid, the gene reporter assay on estrogen-sensitive human breast cancer MCF-7 cells transfected with the ERE-βGlob-Luc-SVNeo plasmid (MELN cells) is applied to test the three pesticides for their possible ER-mediated estrogenic activity. 

Possible additive and/or antagonist effects are also investigated. Moreover, docking simulations provide atomic level insights on the potential binding mode of these molecules to the primary ligand (estrogen) binding site as well as to a peripheral allosteric site which may be responsible for the experimental observed additive effect of the pollutant with the endogenous ligand.

## 2. Materials and Methods 

### 2.1. Materials

All reagents are analytically pure by purchase from Sigma-Aldrich (St. Louis, MO, USA). Stock solutions of chemical compounds were prepared in absolute ethanol or dimethyl sulfoxide (DMSO). Before each experiment, the test sample was diluted into a fresh buffer solution, and the final organic solvent concentration was less than 0.1%. The recombinant human aromatase (Aro) and the human recombinant cytochrome P450 reductase (hCPR) were expressed and purified as previously described [39,49].

### 2.2. ELISA Assay

An estrone direct competitive ELISA kit (BioVendor, Brno, Czech Republic) was used to evaluate the effect of pesticides on aromatase activity. Different reaction mixtures were set up by mixing 5 nM Aro, 5 nM hCPR, 0.5 mM NADPH, 50 nM androstenedione, and three concentrations of pesticides (500, 1000, and 1500 nM) in a 100 mM potassium buffer (KPi) containing 20% glycerol, 1 mM β-mercaptoethanol at pH 7.0. Reactions were carried out for 10 min at 30 °C, heat-inactivated for 10 min at 90 °C, and centrifuged for 10 min at 11,000 rpm. After centrifugation, the supernatant was diluted 1:8 in the Calibrator A provided by the ELISA kit and the product estrone quantified performing ELISA according to the manufacturer’s instructions. Reactions in the presence of anastrozole or without hCPR were used as negative controls. The concentration of estrone was extrapolated from a calibration curve with known concentrations of estrone.

For the experiment where the catalytic parameters were derived, four substrate concentrations were applied in the reaction mixture (ranging from 25 to 250 nM) in the absence and presence of 1000 and 5000 nM of glyphosate.

### 2.3. Computational Studies

In order to explain the molecular terms for the action of glyphosate on the aromatase enzyme we docked it into the two possible allosteric sites previously identified [50]. Docking has been performed with the GLIDE software, release 2020-1 (Schrödinger, LLC, New York, NY, USA) using the single-precision protocol [51]. The two neonicotinoids, thiacloprid and imidacloprid, were instead docked into the ERαs active site, using as a starting structure the crystal structure of 17-β-estradiol (EST)-bound ERα dimer (PDB id: 1qku) [52]. In this structure, we have searched for putative allosteric pockets using the SiteMap algorithm [53].

In order to account for its flexibility of the receptor and since is flexibility resulted to be of paramount importance for the identification of novel allosteric inhibitors [54], we performed classical Molecular Dynamics (MD) simulations on the complex with the aromatase enzyme. We employed as a starting structure of our simulation the equilibrated enzyme model which was embedded in a mimic of a membrane bilayer by using the CHARMMGUI webserver [55]. This consisted of POPC (1-palmitoyl-2-oleoyl-sn-glycero-3-phosphocholine) and 6 wt% of cholesterol (CHL) in order to mimic the endoplasmic reticulum membrane. Physiological protonation states were calculated with the webserver H++ [56]. Asp309 was considered in its neutral form consistently with other literature studies [57]. The glyphosate molecule was considered in the most likely protonation state at physiological pH. According to literature data, the first protonation of the molecule occurs on its phosphate group [58].

The Parm99SB AMBER force field (FF) [59,60] and lipid14 FF [61] were used for the protein and the lipids, respectively.

The Shahrokh et al. parameters were used for the heme moiety and Cys437 [62]. Simulations were done in the presence of the substrate androstenedione (ASD) in the active site and of glyphosate in the allosteric pockets for which the general Amber FF (GAFF) was employed [63]. For the organic ligands the electrostatic potential (ESP) charges [64] were calculated by performing geometry optimization of the substrates at the Hartree-Fock level of theory using a 6-31G* basis set with the Gaussian 09 software (Gaussian Inc., Wallingford, CT, USA) [65]. These were later transformed in RESP charges by using the Antechamber tool [66].

The system was then explicitly solvated using the TIP3P water model, leading to a total of 131,454 atoms. Topology, built with AmberTools 18, was later converted in a GROMACS format using the acpype algorithm [67]. MD simulations were performed with GROMACS 5.0.4 [68]. An integration time step of 2 fs was used and all covalent bonds involving hydrogen atoms were constrained with the LINCS algorithm. The Particle Mesh Ewald algorithm [69] was used in order to account for electrostatic interactions. Simulations were done in the isothermal-isobaric NPT ensemble, at a temperature of 300 K, using a velocity-rescaling thermostat [70]. Preliminary energy minimization was done with the steepest descend algorithm.

An initial equilibration of the membrane was performed for 100 ns with the protein atoms harmonically restrained with a force constant of 1000 kJ mol^−1^ nm^−2^, reaching a constant value (92 × 92 × 151 Å^3^) of the simulation box size. Constraints were then slowly released, and the system was thermalized to the target temperature of 300 K in about 10 ns. Then, the aromatase in complex with glyphosate was relaxed by performing a 100 ns MD simulation rescaling the motion of the center of mass of aromatase and the ligand, followed by an unbiased 100 ns MD simulation.

### 2.4. MELN cell Culture

MELN cells were provided by Dr. P. Balaguer (INSERM, Montpellier, France). They are estrogen-sensitive human breast cancer cells (MCF-7) transfected with the ERE-βGlob-Luc-SVNeo plasmid (ERE-*β*Glob-Luc-SVNeo) [71,72]. The integrated plasmid contains a luciferase reporter gene, the estrogen-responsive elements (ERE) and an antibiotic resistance selection gene (SVNeo). MELN cells were cultured at 37 °C and 5% CO_2_ in Dulbecco’s Modified Eagle’s Medium Nutrient Mixture F12-Ham (DMEM-F12), supplemented with phenol red, fetal bovine serum (FBS) (5% v/v), l-glutamine (4 mM), penicillin-streptomycin (100 U/mL–100 µg/mL), and G418 (1 mg/mL).

### 2.5. MELN Gene Reporter Assay

The assay was carried out as described by Balaguer et al., 1999 [73] with slight modifications [74]. For three days the cells were adapted to a test medium: DMEM-F12 without phenol red and supplemented with dextran-coated charcoal-treated FBS (5% v/v), l-glutamine (4 mM), and penicillin-streptomycin (100 U/mL-100 µg/mL). Then, the cells were seeded at a density of 40,000 cells/well, in 96-well plates (100 μL/well). After 24 h, the test medium of each well was replaced with a test medium containing pesticides (100 μL/wells), and the cells were incubated for 16 h. After the incubation, the luciferase activity was assessed adding 100 μL/well of the One Glo Reagent (One-Glo Luciferase Assay System, Promega, Madison, USA), mixing (5 min) and measuring the luminescence of each well by a luminometer (Infinite Reader M200 Pro, Tecan, Männedorf, Switzerland). 

The stock solutions of thiacloprid and imidacloprid were prepared in DMSO, while the stock solution of glyphosate was prepared in a test medium. The stock solutions were stored at −20 °C and, shortly before exposure, different concentrations of pesticides were prepared in a test medium (glyphosate and imidacloprid: From 10^−8^ to 10^−3^ M; thiacloprid from 10^−8^ to 5 × 10^−4^ M, due to lower solubility). The final DMSO concentration was less than 0.1%. Cells exposed to the test medium were used as a negative control and five concentrations of E2 (from 10^−12^ to 10^−8^ M) were tested to obtain a standard positive curve of the reference compound (E2). 

The estrogenic activity was calculated as the ratio of the activity induced by the treatment over the activity induced by the positive control with 17-βestradiol (E2). It was expressed in percentage considering the relative luciferase activity of E2 (10^−8^ M) as 100%. Since all experiments were performed in quadruplicate (four wells for each experimental condition), the estrogenic activity was expressed as the mean and standard deviation of four values. The estrogenic activity of pesticides was also evaluated by the determination of the relative potency of each pesticide in comparison with the reference compound (E2) and it was expressed as the E2 equivalency factor (EEF) [75]. The EEF was calculated using the concentrations of E2 and pesticides at which 50% of biological effect is achieved (EC50) through the formula: EEF = E2 EC50/pesticide EC50.

Three concentrations of pesticides (10^−5^, 2.5 × 10^−4^, and 5 × 10^−4^ M) were also tested: In combination with an ER-antagonist (tamoxifen 10^−6^ M), in order to confirm whether the observed effects were due to the ER activation, and in combination with E2 (10^−10^ M), in order to investigate the interaction between pesticides and E2 in MELN cells. The estrogenic activity of these treatments was expressed as relative luciferase activity and it was calculated as percentage of activity induced by the treatment with respect to the activity induced by the E2 10^−10^ M (relative luciferase activity of E2 10^−10^ M = 100%). The stock solutions of E2 and tamoxifen were prepared in ethanol and stored at −20 °C.

### 2.6. Data Analysis

Statistical analysis was performed using IBM SPSS Statistics 25.0 (IBM, Armonk, USA). The EC50 of E2 and pesticides was calculated by dose–response curves, which were estimated through a probit regression between the relative luciferase activity and Log transformed-concentrations of E2 or pesticides.

Data collected with the MELN gene reporter assay were not normally distributed, so the non-parametric Kruskal-Wallis test followed by the post-hoc Dunnett test was used to assess significant differences among the different experimental conditions. The differences were considered significant with *p*-value < 0.05.

## 3. Results

### 3.1. Effect of Pesticides on Aromatase Activity

In order to study the effect of pesticide compounds on aromatase activity, a direct competitive estrone ELISA was performed using the purified cytochrome P450 reductase (CPR), as an electron donor from NADPH, and aromatase. The aromatase activity was evaluated by measuring the estrone production in the absence and presence of three different concentrations of pesticides (0.5, 1, and 5 μM). As a control, anastrozole, a known aromatase inhibitor, was applied at a concentration of 1000 nM and the residual aromatase activity detected was 0.7%. As can be seen in Table 1, glyphosate partially reduced the aromatase activity at the concentrations tested. The enzyme activity decreased with the increase of glyphosate concentration. When adding 5 μM of glyphosate, the residual aromatase activity was 36%. Unlike glyphosate, imidacloprid and thiacloprid did not inhibit the enzyme activity (Table 1).

### 3.2. Effect of Glyphosate Concentration on Aromatase Activity 

The effect of glyphosate on aromatase activity was further studied by exploring the concentration range of glyphosate applied from 50 to 1500 nM. Such concentrations of glyphosate were selected since they resemble the ones detected in human urine samples [76]. The experiment was carried out by the ELISA assay at the concentration of 50 and 400 nM androstenedione, respectively. The two different concentrations were chosen on the basis of the kinetic parameters of aromatase: The first one (50 nM) is close to the enzyme K_M_ and the second one (400 nM) is saturating the enzyme (see next paragraph).

**Figure 1:**
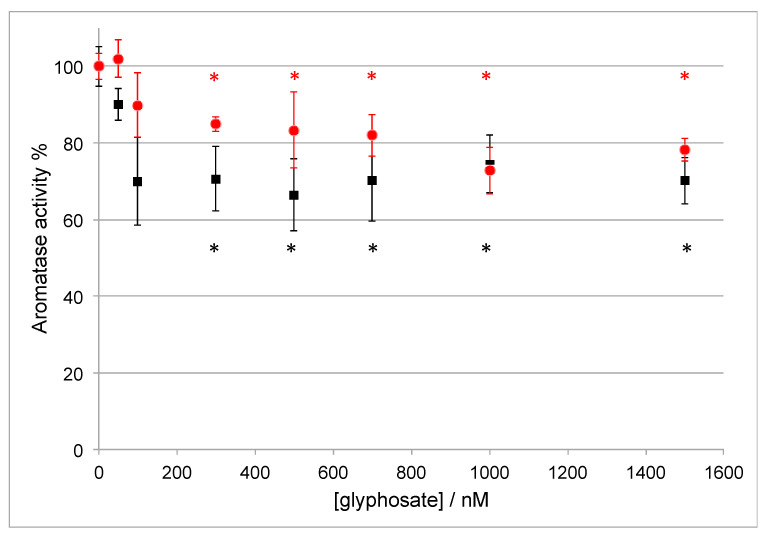
Aromatase activity in the presence of different concentrations of glyphosate and 50 nM (black squares) or 400 nM (red circles) of the substrate androstenedione. Statistical significance *: *p*-value < 0.05 versus C^+^.

As shown in Figure 1, the activity of Aro is inhibited by 30% when the glyphosate concentration is ≥1000 nM. However, when the substrate concentration is 50 nM (black squares in Figure 1), the maximal inhibitory effect is already achieved when the glyphosate concentration is 100 nM. Therefore, the inhibitory effect of glyphosate strongly depends on the substrate concentration and it is only partial, indicating that this compound can be considered as a weak inhibitor.

### 3.3. Effect of Glyphosate on the Catalytic Parameters of Aro

**Figure 2:**
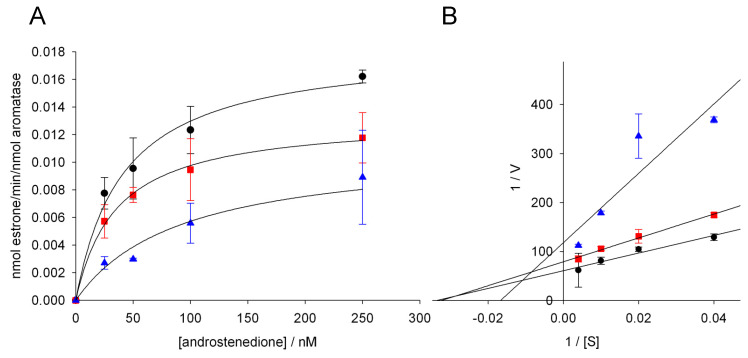
(**A**) Michaelis-Menten plots and (**B**) Lineweaver-Burk linearization for aromatase activity in the absence (black circles) and presence of 1000 nM (red squares) and 5000 nM (blue triangles) of glyphosate. In Panel (**A**), the data were fitted to the Michaelis-Menten equation using the Sigma Plot software to obtain the kinetic parameters.

In order to investigate the mechanism of aromatase inhibition by glyphosate, the kinetic parameters of the enzyme were evaluated using the estrone ELISA assay in the absence and presence of two different concentrations of the pesticide (1000 and 5000 nM). Different substrate concentrations were applied and the product formation rate was plotted as a function of the substrate concentration (Figure 2A). The plot showed hyperbolic trends and the catalytic parameters, shown in Table 2, were obtained by fitting the experimental data to the Michaelis-Menten equation.

When glyphosate is not present, the resulting K_M_ and V_max_ are 41.3 ± 8.2 nM and 0.018 ± 0.001 min^−1^, respectively. When 1000 nM of glyphosate was added, the V_max_ value was significantly decreased whereas the K_M_ value was not significantly affected. Interestingly, when the glyphosate concentration was increased to 5000 nM, both K_M_ and V_max_ were affected. Compared to the reaction without glyphosate, K_M_ was increased by 2.2 folds, while V_max_ was decreased to 0.011 ± 0.002 min^−1^ (Table 2).

The kinetic parameters show that the type of inhibition of aromatase by glyphosate depends on the herbicide concentration applied. Indeed, when using 1000 nM of glyphosate, the V_max_ was decreased while K_M_ did not change, indicating a non-competitive inhibition mechanism, meaning that, at this concentration, glyphosate does not compete with the substrate and binds to a site different from that where the substrate binds. When the concentration of glyphosate was increased to 5000 nM, both K_M_ and V_max_ were affected, and the Lineweaver-Burk plot shows a trend typical of a mixed inhibition mechanism (Figure 2B). Mixed inhibition is considered a more general case of non-competitive inhibition, in which the inhibitor exhibits unequal affinity for the free enzyme and for the enzyme-substrate complex.

### 3.4. Molecular Dynamics Simulations on Aromatase 

**Figure 3:**
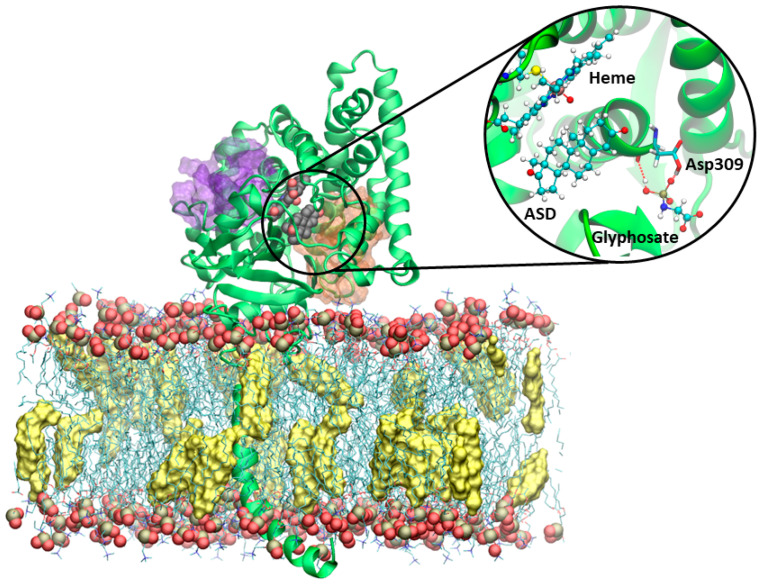
Representative structure of aromatase embedded in a POPC (1-palmitoyl-2-oleoyl-sn-glycero-3-phosphocholine) membrane, with phosphorous and oxygen atoms shown as tan and red van der Waals (vdw) spheres, and cholesterol (yellow surface) membrane. Sites 1 and 2 are shown as orange and purple transparent surfaces, respectively. The heme and androstenedione (ASD) are displayed in a vdw representation. The protein is shown as green new cartoons. The inset reports a close view of structure of aromatase in complex with glyphosate, as obtained from the most representative cluster of the molecular dynamics simulation trajectory. The heme moiety, ASD, and glyphosate are depicted in balls and sticks. The key catalytic residue Asp309, lining the binding cavity, is shown in licorice and colored by the atom name.

Grounding on recent evidence demonstrating the existence of allosteric binding sites [50] and their possible exploitation for a non-competitive/mixed inhibition mechanism [54] we docked glyphosate into the two allosteric cavities. Namely, we docked it to Site 1, which lies along the most relevant access channel to the enzyme active site [77] and to Site 2, which instead lies at the interface with the cytochrome P450 reductase (CPR), supplying the electrons necessary for catalysis (Figure 3) [78]. In the docking pose in Site 1 and during MD simulations glyphosate engages a salt bridge interaction with its phosphate group and Arg192, as well as the formation of a hydrogen (H)-bond between Gln218 and the carboxylic group of the pesticide. Most importantly, the phosphate group of glyphosate makes up to two simultaneous H-bonds with Asp309 (Figure 3), which normally is engaged in stabilizing the binding of aromatase substrates.

Due to these interactions the molecule remains stably bound in the pocket for the whole MD simulation, in line with its inhibitory activity in the µM range. Remarkably, it was recently suggested that the binding of a small molecule in Site 1 triggered the displacement of the water molecule needed for the catalytic activity, which are normally H-bonding with the Asp309 and Arg192 residues, both being critical residues for the catalytic activity [41,57], thus inhibiting estrogen biosynthesis. Conversely, the docking pose obtained in Site 2, did not establish any relevant H-bond/salt bridge. As a result, the glyphosate dissociated from the pocket within the first few ns of MD simulations.

### 3.5. Detection of Estrogenic Activity with the MELN Gene Reporter Assay

The MELN gene reporter assay was carried out to evaluate the estrogenic activity of glyphosate, imidacloprid, and thiacloprid on MELN cells. In this study, different concentrations of each pesticide were tested. Concentrations similar to the pesticide levels measured in human urine [76] were selected as the lowest concentrations (10^−8^ and 10^−7^ M), while concentrations up to 10^−3^ M were selected as the highest concentrations, in order to assess the effect induced by each pesticide in a wide range of concentrations.

**Figure 4:**
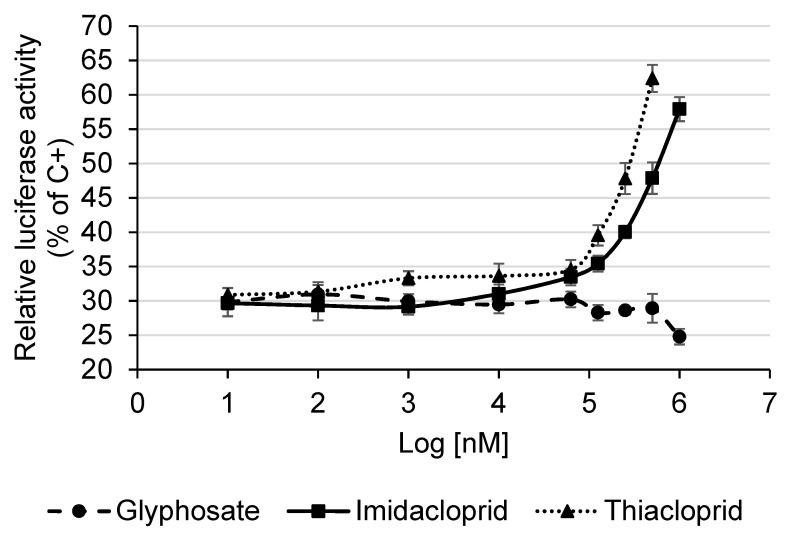
Estrogenic activity of pesticides measured with the MELN gene reporter assay. Data are expressed as means and standard deviations of the relative luciferase activity (% of C+, E2 10^−8^ M). The relative luciferase activity of the C+ is 100.0 ± 5.9%, while the relative luciferase activity of the negative control (test medium) is 30.5 ± 1.1%.

Our results showed that glyphosate did not increase the relative luciferase activity with respect to the negative control; therefore no estrogenic activity was detected testing this pesticide on MELN cells (Figure 4). On the contrary, the highest concentrations of glyphosate induced a small decrease of the relative luciferase activity, which may be due to a toxic effect of the pesticide on cells. 

The null estrogenic activity induced by glyphosate was confirmed also by the exposure of cells to glyphosate in combination with tamoxifen (ER-antagonist) or with E2. Indeed, the wells treated with glyphosate and tamoxifen showed a relative luciferase activity equal to the wells treated with tamoxifen alone, and the wells treated with glyphosate and E2 showed a relative luciferase activity equal to the wells treated with E2 alone (data not shown). These results suggest that glyphosate does not interfere with the binding between ER and E2.

Regarding the neonicotinoid pesticides, in the present study, imidacloprid and thiacloprid significantly increased the relative luciferase activity with respect to the negative control, starting from 6.3 × 10^−5^ M (Log[nM] = 4.79) and 10^−6^ M (Log[nM] = 3), respectively (Kruskal-Wallis test followed by the post-hoc Dunnett test, *p* < 0.05). Since both the neonicotinoid pesticides induced a dose-dependent increase of the relative luciferase activity, in particular from 10^−4^ M (Log[nM] = 5) to the highest tested dose (Figure 4), a significant estrogenic activity of these two pesticides was detected on MELN cells.

The estrogenic activity of imidacloprid and thiacloprid was also quantitatively evaluated by the estimate of the concentrations of E2 and pesticides at which 50% of biological effect is achieved (EC50) and the E2 equivalency factor (EEF). The EC50 of E2 and pesticides was calculated by dose-response curves whereas the EEF was calculated through the formula: EEF = E2 EC50/pesticide EC50. The EC50 of imidacloprid and thiacloprid was 1.0 × 10^−2^ M (IC 95% 1.7 × 10^−3^–2.2 × 10^−1^ M) and 1.5 × 10^−3^ M (IC 95% 2.5 × 10^−4^–3.6 × 10^−2^ M), respectively, while the EEF was 5.4 × 10^−10^ (IC 95% 3.3 × 10^−9^–2.5 × 10^−11^) and 3.7 × 10^−9^ (IC 95% 2.2 × 10^−8^–1.5 × 10^−10^), respectively.

**Figure 5:**
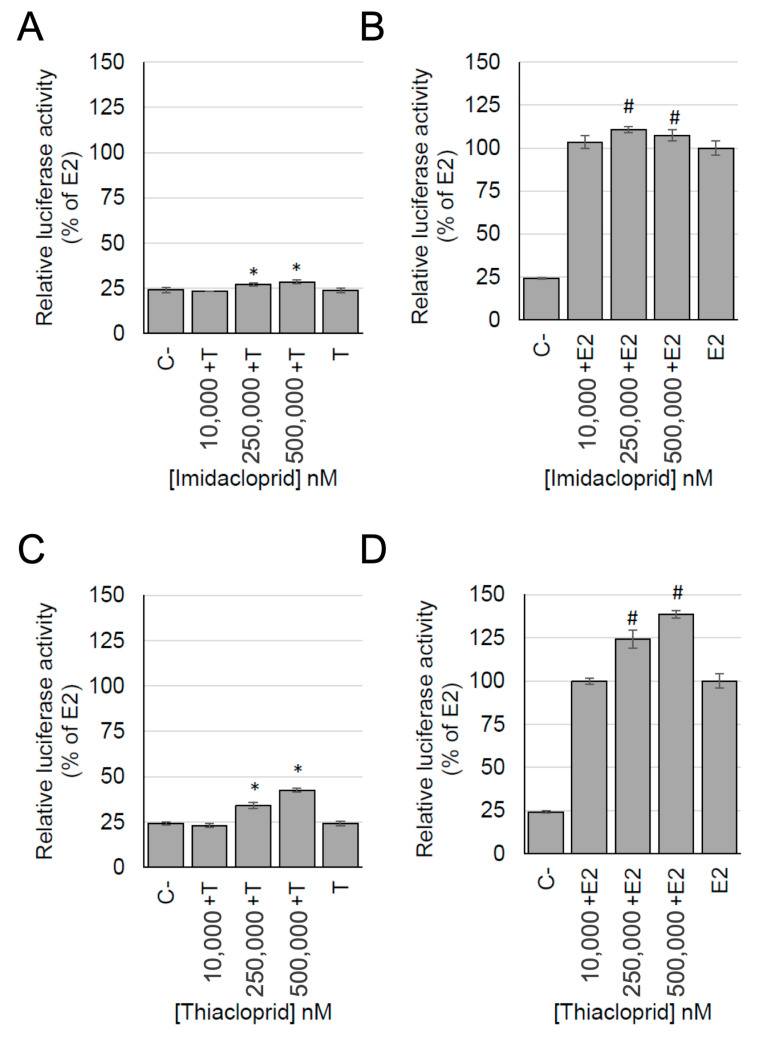
Estrogenic activity, measured with the MELN gene reporter assay, of imidacloprid (**A**,**B**) and thiacloprid (**C**,**D**) in combination with E2 (10^−10^ M) (**B**,**D**) or in combination with tamoxifen (10^−6^ M) (**A**,**C**). Data are expressed as the relative luciferase activity (% of E2 10^−10^ M). C−: Negative control; E2: E2 10^−10^ M; T: Tamoxifen 10^−6^ M. * *p* < 0.05 vs. C−; # *p* < 0.05 vs. E2; Kruskal-Wallis test followed by the post-hoc Dunnett test.

The exposure of cells to imidacloprid and thiacloprid in combination with tamoxifen confirmed that the estrogenic activity of the two pesticides was induced by the activation of ER. Indeed, the wells treated with the neonicotinoid pesticides and tamoxifen showed a relative luciferase activity that was lower compared to the wells treated with these pesticides alone. Furthermore, the relative luciferase activity of wells treated with the neonicotinoid pesticides and tamoxifen was similar to the relative luciferase activity measured in the negative control (Figure 5A,C).

Finally, the exposure of cells to imidacloprid and thiacloprid in combination with E2 induced an increase of the relative luciferase activity with respect to the E2 alone (Figure 5B,D). The increase was slight for imidacloprid while it was stronger for thiacloprid, suggesting a possible additive effect exerted by these pesticides in combination with E2.

### 3.6. Docking Calculation on ERα

**Figure 6:**
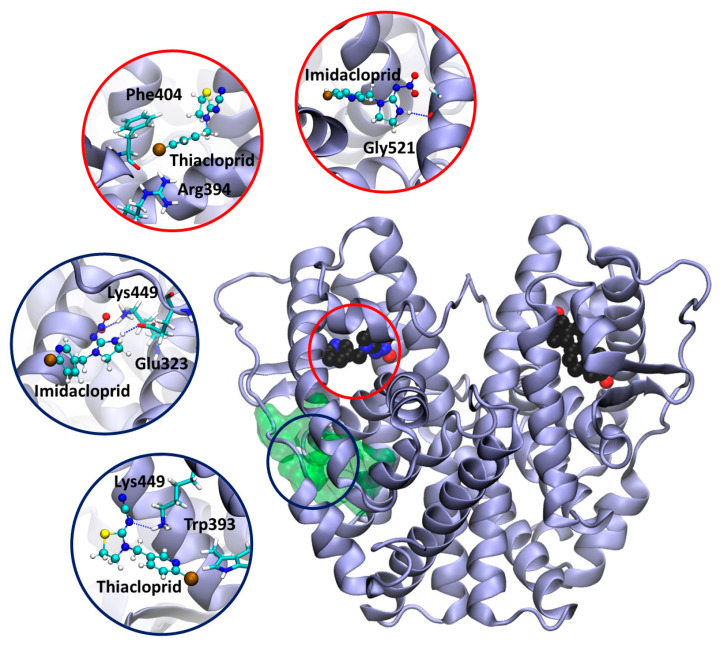
Model of estrogen receptor α dimer (PDB ID 1qku [52]) in complex with the neonicotinoids, imidacloprid and thiacloprid. The allosteric pocket is shown as a green transparent surface. Imidacloprid, thiacloprid, and 17-β-estradiol are displayed in a van der Waals representation and colored by the atom name. The protein is shown as violet new cartoons. The insets report a close view of docking poses of imidacloprid and thiacloprid inside the estrogen binding site (red circles) and onto the newly identified allosteric pocket (dark blue circles). The ligand and the residues establishing the most important interactions are depicted in balls and sticks and licorice representations, respectively, and colored by the atom name.

In order to provide a rationale for the estrogenic activity exerted by the two neonicotinoids, thiacloprid and imidacloprid, we have performed docking calculations on ERα. First, the two molecules were docked into the ERαs estrogen binding site, using the crystal structure of 17-β-estradiol bound to the ERα dimer (PDB id: 1qku) [52]. Both neonicotinoids fit inside the estrogen binding pocket (Figure 6). In particular, imidacloprid forms a H-bond with the backbone of Gly521, while in thiacloprid, the Cl atom makes halogen bonds with the guanidinium group of Arg394 and the aromatic rings of Phe404 and Trp393. Halogen bonds are attractive interactions between the electrophilic region associated with the Cl halogen atom and the nucleophilic regions of the surrounding protein residues [79].

Next, in order to disclose if and how the two neonicotinoids exert an additive effect to estrogen binding, by occupying an allosteric cavity, we looked for the presence of druggable allosteric pockets in the protein. Interestingly, a high-ranking binding pocket was found in the proximity of the estrogen binding site of one monomer of ERα (Figure 6). The docking calculation performed on this pocket, strikingly revealed that imidacloprid can H-bond with Lys449 and Glu323. As well thiacloprid H-bonds with Lys449 and Trp393.

## 4. Discussion

In this study, the effect of three pesticides on two key targets of the endocrine system was evaluated using a combination of experimental and in silico methods that allowed an investigation at the molecular level. 

The first target considered was aromatase, the key enzyme for estrogen production that resulted in being partially inhibited by glyphosate. The increase obtained in the aromatase activity in the presence of imidacloprid and thiacloprid, even if not statistically significant, can be interesting to be further investigated. Indeed, these two compounds could directly act on aromatase as allosteric activators or they could exert their action indirectly on CPR that has an essential role in catalysis and acts as an effector on human aromatase conformation [80,81].

Glyphosate is the most widely used active compound among herbicides and was already reported to affect aromatase expression and activity in cells [33,35]. The impact for human health has already been demonstrated: Indeed, an alteration in androgens/estrogens balance due to a lower aromatase expression as a consequence of the glyphosate presence changed the sperm nuclear quality impacting mammalian reproduction [82].

However, the direct interaction between glyphosate and the enzyme together with the inhibition mechanism was not reported yet. In this study, the ELISA assay revealed that the inhibition of aromatase by glyphosate is partial and weak and strongly depends on the substrate concentration. Moreover, at the lower concentration of glyphosate tested (1 μM), the inhibition was found to be non-competitive, while at the higher concentration used (5 μM), the inhibition turned into a mixed inhibition mode. These data suggest that glyphosate binds to an allosteric site both when the enzyme is free and in complex with the substrate. Classical MD simulations supply a structural model for glyphosate binding to aromatase, explaining at the atomic-level that this pollutant may exert its inhibitory activity at a low concentration by binding to the allosteric Site 1 previously identified by Magistrato et al. [50] and demonstrated to bind small-molecules inhibitors [54]. When the concentration increases, a mixed inhibition is observed again compatible with the presence of an allosteric site. The binding of glyphosate probably becomes stronger or another allosteric site is occupied by the inhibitor. However, MD simulations showed that glyphosate did not stably bind to the second allosteric pocket (Site 2) identified in previous studies.

The second important target studied was the estrogen receptor that is responsible for estrogen binding and signal transduction in cells. MCF-7 cells, stably transfected with an estrogen-regulated luciferase gene (MELN cells), were used to assess the estrogenic activity of the pesticides. Glyphosate induced no estrogenic activity on MELN cells, moreover glyphosate did not change the effect induced by tamoxifen or E2. These results are in accordance with the results of the studies of Kojima et al., 2004 [46] and Gasnier et al., 2009 [35], who did not find any agonistic or antagonistic effect of glyphosate on ER. On the contrary, our results are different than the results of Thongprakaisang et al., 2013 [44] and Mesnage et al., 2017 [45] who found a significant increase of ER-induced estrogenic activity. A possible explanation of this discrepancy could be that in our study the estrogenic activity was evaluated on MELN cells while the other two studies applied T47D-KBluc cells, which might be more sensitive to the glyphosate activity than MELN cells. Moreover, our results are consistent with the results of the Endocrine Disruptor Screening Program (EDSP) conducted by the United States Environmental Protection Agency (US EPA, 2015) which concluded that “there is no convincing evidence of a potential interaction with the estrogen pathway for glyphosate”.

In the present study, both imidacloprid and thiacloprid induced a dose-response estrogenic activity mediated by the ER activation, in particular starting from 10^−4^ M. A consistent result was found in the study of Kojima et al., 2004 [46] in which no estrogenic activity was detected exposing transfected-cells to imidacloprid at concentrations lower than 10^−5^ M; a contradicting result was found by Westlund and Yargeau, 2017 [48], who did not find any estrogenic activity testing thiacloprid at concentrations comparable to ours. The conflicting results of thiacloprid should be interpreted considering the different cell models applied in our study with respect to the study of Westlund and Yargeau, 2017 (i.e., the present study was performed on mammalian cells while the previous one on yeast cells) [48]. Indeed, yeast cells are characterized by a different membrane permeability, transport proteins, and signal transduction pathways with respect to mammalian cells which may have influenced the results [42], as reported before also for other nuclear receptors [83].

The estrogenic activity of imidacloprid and thiacloprid was observed at concentrations higher than the pesticide levels measured in human biological samples [76,84]. Although such high levels of pesticides are not found in human biological samples, low doses of these pesticides should not be considered harmless. Indeed, in biological fluids these pesticides may be present in combination with other EDCs with a similar action mode and, thus, these pesticides together with other EDCs might cause an overall estrogenic effect. Moreover, these high concentrations of pesticides are similar to levels in some environmental matrices, where they could cause adverse health effects on wildlife animals. For example, in guttation water or morning dew of plants, imidacloprid was found to be present at a concentration up to 346 mg/L [85].

Interestingly, the results of the present study showed that, when the ER-antagonist tamoxifen was added to these pesticides, the estrogenic activity was still higher than the negative control. This result can be explained by the competition between each pesticide and tamoxifen for ER binding. Moreover, in the presence of high concentrations of imidacloprid and thiacloprid, an additive effect with E2 was also observed. The docking calculation suggested that these two neonicotinoids may bind to both the orthosteric and allosteric pockets of ERα, suggesting a putative mechanism to rationalize their observed estrogenic activity.

## 5. Conclusions

In conclusion, this study provides further evidence about the action of some pesticides as endocrine disrupting chemicals (EDCs) targeting important proteins of the endocrine system. In particular, it shows that the inhibitory effects of the three compounds tested on aromatase are partial and their estrogenic effects occur at relatively high concentrations. However, possible additive estrogenic effects with the physiological hormone 17-βestradiol are present. Furthermore, it has to be taken into account that pesticides are usually introduced in the environment with their co-formulants that can be also biologically active as EDCs. Previous studies already showed that aromatase is inhibited by co-formulants of glyphosate-based herbicides [33,86]. Moreover, more studies are needed to investigate a possible additive effect of different pesticides that can be contemporarily present in the environment.

Our study also provides an integrated approach based on different assays and computational methods that allowed gaining new information about the possible interaction of pesticides and key targets at a molecular level. Such information can be exploited to predict the possible impact of other compounds on estrogen production and signaling in order to develop safer compounds for human health and environment.

## Figures and Tables

**Table 1:** Effect of pesticides on the aromatase activity.

	Pesticide	Relative Activity (%)		
		500 nM	1000 nM	5000 nM
1	Glyphosate	76.6 ± 11.3 ^*^	74.5 ± 7.6 ^*^	36.0 ± 19.5 ^*^
2	Imidacloprid	100.1 ± 8.8	92.9 ± 28.0	120.5 ± 17.6
3	Thiacloprid	100.9 ± 11.9	153.6 ± 56.9	146.9 ± 42.1

Statistical significance *: *p*-value < 0.05 versus positive control (C^+^).

**Table 2:** Kinetic parameters obtained from the fitting of the data in Figure 2A to a Michaelis-Menten curve. The kinetic parameters are calculated for aromatase activity in the absence and presence of 1000 and 5000 nM of glyphosate.

Glyphosate (nM)	K_M_ (nM)	V_max_ (min^−1^)
0	41.3 ± 8.2	0.018 ± 0.001
1000	35.3 ± 3.5	0.013 ± 0.001 ^*^
5000	92.3 ± 20.7 ^*^	0.011 ± 0.002 ^*^

Statistical significance*: *p*-value < 0.05 versus the values obtained in the absence of glyphosate.
